# Porcine peritoneal macrophages are susceptible to porcine reproductive and respiratory syndrome virus infection

**DOI:** 10.3389/fmicb.2024.1505900

**Published:** 2024-12-11

**Authors:** Kassandra Durazo-Martinez, Jayeshbhai Chaudhari, Sushmita Kumari, Hiep L. X. Vu

**Affiliations:** ^1^Department of Animal Science and Nebraska Center for Virology, University of Nebraska-Lincoln, Lincoln, NE, United States; ^2^School of Veterinary Science and Nebraska Center for Virology, University of Nebraska-Lincoln, Lincoln, NE, United States

**Keywords:** porcine peritoneal macrophages, PRRSV, tropism, CD163, swine virus

## Abstract

Previous studies have suggested that porcine peritoneal macrophages (PPMs) are resistant to PRRSV infection, whereas porcine alveolar macrophages (PAMs) are highly susceptible. This contrast is intriguing, as both cell types belong to the same monocyte/macrophage family. The current study aimed to investigate the host factors contributing to the differing susceptibility of PPMs and PAMs to PRRSV infection. We found that PPMs exhibit a higher frequency of CD14^+^ cells compared to PAMs, suggesting a more immature macrophage phenotype in PPMs. Importantly, PPMs expressed both CD163 and CD169, the key receptors for PRRSV entry, although the frequency and intensity of CD163 and CD169 expression were lower in PPMs than in PAMs. Despite these differences, PPMs were susceptible to both PRRSV-1 and PRRSV-2 isolates. Notably, PPMs susceptibility increased 10-fold when the cells were cultured for 1 day before infection. PRRSV infection in PPMs was dependent on CD163, as pretreatment with an anti-CD163 antibody significantly reduced infection. Overall, our results demonstrate that PPMs are susceptible to PRRSV infection, thereby expanding the understanding of PRRSV tropism.

## 1 Introduction

Porcine reproductive and respiratory syndrome virus (PRRSV) is the causative agent of a panzootic disease that has caused significant economic losses to the pig industry (Neumann et al., 2005). The virus belongs to the family *Arteriviridae*, together with Equine Arteritis Virus, Lactate Dehydrogenase Elevating Virus, and Simian Hemorrhagic Fever Virus (Brinton et al., 2021). PRRSV is further classified into two species: *Betaarteriviruses suid 1*(*formerly* PRRSV-1) and *Betaarteriviruses suid 2* (*formerly* PRRSV-2) (Brinton et al., 2021). PRRSV has a narrow host and cell tropism. Pigs are the only known natural host of PRRSV and cells of the myeloid lineage are the primary targets of the virus (Duan et al., 1997a).

Early studies suggested that only certain subpopulations of swine macrophages are susceptible to PRRSV, although the specific markers of these subpopulations remain unclear (Duan et al., 1997b). The cell’s origin appears to influence its susceptibility. For example, porcine alveolar macrophages (PAMs) are highly susceptible to PRRSV infection, while peripheral blood monocytes (BMo) and peritoneal macrophages (PPMs) are resistant (Duan et al., 1997b). Similarly, monocyte-derived dendritic cells (MoDCs) and bone marrow-derived dendritic cells (BmDCs) are fully permissive to infection, whereas *bona fide* dendritic cells (DCs) and plasmacytoid DCs are not (Loving et al., 2007; Wang et al., 2007; Chang et al., 2008; Flores-Mendoza et al., 2008; Park et al., 2008; Silva-Campa et al., 2009; Reséndiz et al., 2018). The maturation stages of monocytes and macrophages also influence their susceptibility. Although PAMs are highly susceptible to PRRSV infection, their susceptibility further increases after *ex vivo* culture for 24 h (Duan et al., 1997b). Likewise, BMo become susceptible to PRRSV after *ex vivo* culture (Duan et al., 1997b). Treatment with interleukin-10 or dexamethasone also renders BMo susceptible to PRRSV infection (Singleton et al., 2018). Interestingly, PPMs remain resistant to PRRSV infection even after *ex vivo* culture (Duan et al., 1997b).

While multiple cellular receptors have been identified as being involved in PRRSV infection, CD163 is well-established as the key receptor (Calvert et al., 2007). The ectopic expression of CD163 alone in nonsusceptible cells is sufficient to render them susceptible to PRRSV. On the other hand, treatment of susceptible cells with anti-CD163 antibodies abrogates their susceptibility (Calvert et al., 2007). Gene-edited pigs lacking CD163 are completely resistant to PRRSV, further demonstrating the crucial role of this receptor (Whitworth et al., 2014; Whitworth et al., 2016; Yang et al., 2018).

CD169, or sialoadhesin, also contributes to cellular susceptibility to PRRSV. Pretreatment of susceptible cells with an anti-CD169 antibody inhibits PRRSV infection (Duan et al., 1998a; Duan et al., 1998b; Vanderheijden et al., 2003). The absence of CD169 expression in PPMs is thought to be the reason for their resistance to PRRSV (Duan et al., 1997b; Duan et al., 1998a). However, gene-edited pigs lacking CD169 remain fully susceptible to PRRSV infection, indicating that this receptor is not essential (Prather et al., 2013). Further analysis revealed that CD169 facilitates the attachment and entry of virus particles into macrophages but does not support the uncoating process (Vanderheijden et al., 2003). Nonetheless, cells co-transfected with CD169 and CD163 exhibit greater susceptibility to PRRSV than those transfected with CD163 alone, which clearly indicates the supportive role of CD169 in PRRSV entry (Vanderheijden et al., 2003; Van Gorp et al., 2008).

The contrasting susceptibility between PAMs and PPMs reported in previous studies (Duan et al., 1997b) is intriguing, given that they are both macrophages. Therefore, we aimed to analyze the cellular markers associated with the maturation stages of macrophages, as well as those involved in PRRSV entry, to better understand the differing susceptibilities observed between PAMs and PPMs. Our analysis revealed that both cell types express CD163 and CD169, the key molecules involved in infection. Upon infection with various PRRSV isolates, we found that PAMs were highly susceptible, as expected. Interestingly, contrary to previous reports, PPMs were also highly susceptible to both PRRSV-1 and PRRSV-2 isolates. Furthermore, pretreatment of PPMs with an anti-CD163 antibody reduced the infection. Overall, our results indicate that PPMs are susceptible to PRRSV, which can be attributed to their expression of CD163.

## 2 Materials and methods

### 2.1 Institutional review board statement

The animals used in this study were housed and handled in accordance with the standard operating procedures approved by the University of Nebraska-Lincoln Institutional Animal Care and Use Committee under protocol number 2310, approved on September 07, 2019.

### 2.2 Cell isolation

PAMs and PPMs were collected from 4- to 5-week-old PRRSV-seronegative pigs. PAMs were isolated via lung lavage, while PPMs were obtained through peritoneal cavity lavage using cold PBS. The collected cells were filtered through a 70 μm mesh to remove clumps and debris, then centrifuged at 350 × *g* for 10 min and resuspended in Roswell Park Memorial Institute (RPMI) 1640 medium supplemented with 10% fetal bovine serum (FBS) and antibiotics (cRPMI; 100 units/mL penicillin, 100 μg/mL streptomycin). After one additional wash in cRPMI, the cells were resuspended in the same medium, counted, and seeded as required for the experiments. PAMs and PPMs were cultured under identical conditions at 37°C and 5% CO_2_. For each experiment, cells from at least three pigs were used.

### 2.3 Reagents and antibodies

The antibodies used for characterization included mouse anti-pig CD169 conjugated to Alexa Fluor 647 (Bio-Rad; clone 3B11/11), mouse anti-human CD14 conjugated to StarBright Violet 610 (Bio-Rad; clone TÜK4), mouse anti-pig CD163 conjugated to PE (Thermo Fisher Scientific; clone 2A10/11), and mouse anti-pig CD172a (Thermo Fisher Scientific; clone BL1H7), detected with a secondary goat anti-mouse IgG Alexa Fluor 488 F(ab’)₂ fragment. All primary antibodies were diluted 1:100, and the secondary antibody was diluted 1:500. Fixation and permeabilization were performed using the Cytofix/Cytoperm kit (BD Biosciences). For virus detection, two monoclonal antibodies specific to the viral N protein were used: SDOW17 (National Veterinary Services Laboratories) and SR-30 conjugated to FITC (Rural Tech Inc.). The goat anti-human CD163 polyclonal antibody (R&D Systems; clone AF1407) was used for the receptor-blocking assay.

### 2.4 Viruses

The PRRSV-1 strain SD0108 (GenBank accession number DQ489311.1) was generously provided by Dr. Fang (University of Illinois Urbana-Champaign) (Fang et al., 2006). The PRRSV-2 strain FL12 (GenBank accession no. AY545985) was recovered from a cDNA clone as previously described (Truong et al., 2004). Both SD0108 and FL12 were propagated in MARC-145 cells. Two PRRSV-2 field isolates, RFLP-144 and RFLP-184, were obtained from serum samples collected at two separate infected farms in Nebraska in 2022. Both field isolates were propagated in PAM cells.

### 2.5 Flow cytometry

Freshly isolated PAMs and PPMs were seeded at a density of 5 × 10^5^ cells per tube and washed twice with FACS buffer (1X PBS supplemented with 4% FBS). The cells were then incubated with anti-CD163, anti-CD169, and anti-CD14 antibodies (all at 1:100 dilution in FACS buffer) for 30 min in the dark at 4°C. Staining for CD172a was performed in a separate tube under the same conditions, followed by incubation with the goat anti-mouse IgG Alexa Fluor-488 antibody (1:500 dilution in FACS buffer). After three additional washes with FACS buffer, the cells were fixed and permeabilized using Cytofix/Cytoperm solution, according to the manufacturer’s instructions.

For the characterization of cells after PRRSV infection, the same protocol for surface staining was followed. After permeabilization, the cells were incubated with the FITC-conjugated anti-N antibody (clone SR-30, 1:100 dilution in Perm/Wash buffer) for 30 min in the dark at 4°C. The cells were analyzed using a CytoFlex cytometer (Beckman Coulter, Fremont, CA, United States), with 30,000 events recorded per sample. Data analyses were conducted using FlowJo software (BD Biosciences, San Jose, CA, United States).

### 2.6 Infection and virus titration

All infections were performed using cells that had been cultured overnight, unless otherwise noted. The cells were inoculated with different PRRSV isolates at a multiplicity of infection (MOI) of 2. After incubating with each virus for 1 h at 37°C, the cells were washed three times and replenished with cRPMI. After 24 h of infection, the cell supernatant was collected for virus titration in PAM cells using the endpoint dilution assay. Viral yield was determined by subtracting the virus titer at time 0 from the virus titer at 24 h.

### 2.7 Indirect immunofluorescent assay

Infected and mock-infected cells were washed twice with 1X PBS, then fixed with cold methanol: acetone (1:1, v/v) for 10 min and air-dried. The cells were rehydrated with 1X PBS before incubating with SDOW17 (1:500 dilution in 1X PBS) for 1 h at room temperature (RT). After three washes with 1X PBS, the cells were incubated for an additional 1 h with the goat anti-mouse IgG Alexa Fluor-488 antibody (1:1,000 dilution in 1X PBS). Following another three washes with 1X PBS, the cell nuclei were stained with DAPI (4′, 6′-diamidino-2-phenylindole; 1:3000 dilution in 1X PBS) for 5 min at RT. After a final wash, fluorescence was observed under an inverted microscope (Nikon Eclipse Ts2R-FL, operated by Nikon NIS Elements, version 5.02).

### 2.8 CD163 blocking assay

The receptor-blocking assay was conducted as previously described (Calvert et al., 2007). PPMs and PAMs were cultured either in adhesion in a 96-well plate or in suspension in a culture tube at a density of 5 × 10^5^ cells overnight. Following incubation, the cells were treated with 10 μg of goat anti-human CD163 polyclonal antibody for 1 h at 37°C before being infected with the PRRSV strain FL12 at an MOI of 2. After 1 h of adsorption at 37°C, the cells were washed three times with RPMI and further cultured in cRPMI. At 24 h post-infection (hpi), virus-infected cells were detected using flow cytometry and immunofluorescence assay (IFA).

### 2.9 Statistical analysis

Statistical analyses were performed in the GraphPad Prism Version 9.5.1 (Graph Pad Software Inc.). All analyses were performed using unpaired t-test analyses corrected by the Bonferroni-Dunn method. *p* > 0.05 = not significant (ns; no shown in graph), * *p* ≤ 0.05, ** *p* ≤ 0.01, *** *p* ≤ 0.001, **** *p* ≤ 0.0001.

## 3 Results

### 3.1 Peritoneal macrophages expresses key receptors for PRRSV infection

**Figure 1:**
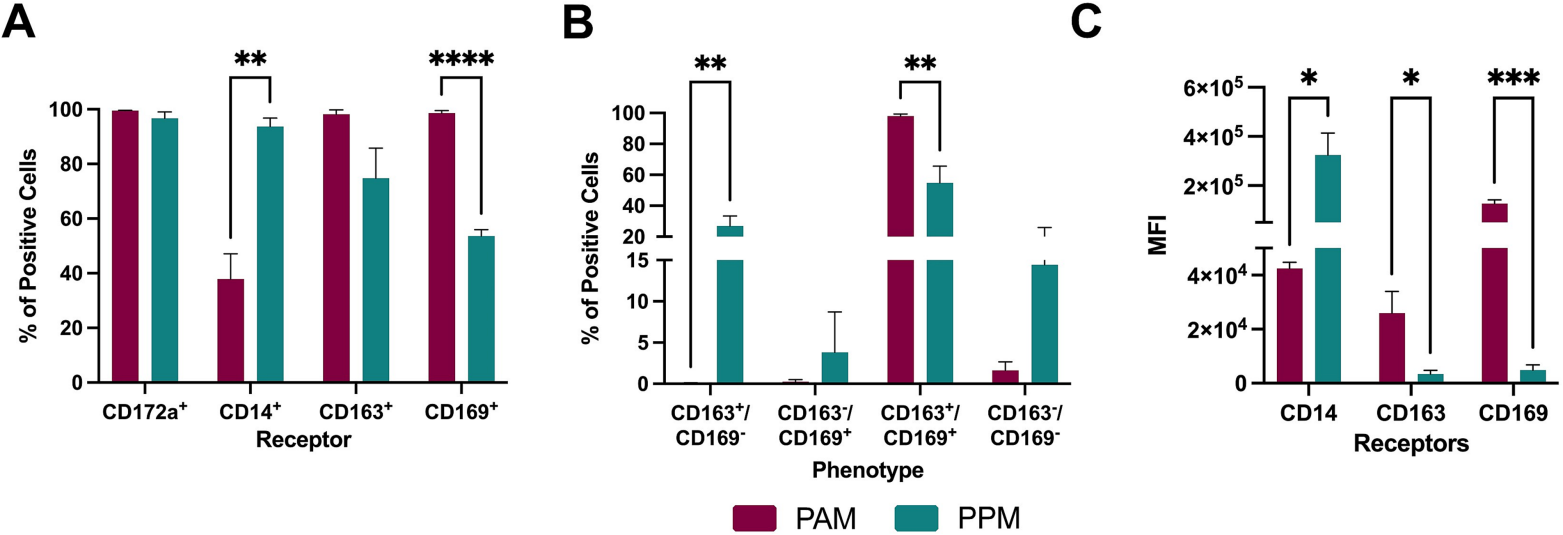
Expression of surface receptors. Freshly isolated PAMs and PPMs were analyzed by flow cytometry for the expression of CD172a, CD14, CD169, and CD163 proteins. **(A)** Percentage of cells individually expressing each of the examined markers. **(B)** Percentage of cells co-expressing CD163 and CD169 receptors. **(C)** MFI of the cell markers. The experiments were conducted using cells collected from three different pigs. * *p* ≤ 0.05, ** *p* ≤ 0.01, *** *p* ≤ 0.001, **** *p* ≤ 0.0001.

We collected PPMs and PAMs samples and assessed the expression of CD172a, a characteristic marker of swine macrophages (Álvarez et al., 2000). Both PPM and PAM samples contained over 90% CD172a^+^ cells, indicating a high frequency of macrophages in both populations (Figure 1A). To further characterize the maturation stage of these macrophages, we examined CD14 expression, a marker that is downregulated in mature macrophages (Mccullough et al., 1999). PPMs had a higher percentage of CD14^+^ cells than PAMs (Figure 1A), suggesting a greater proportion of immature macrophages in PPMs compared to PAMs.

Next, we assessed the expression of two major cellular receptors involved in PRRSV entry: CD163 and CD169. When each receptor was analyzed separately, PAMs had a higher percentage of CD163^+^ and CD169^+^ cells than PPMs, although it did not reach statistical significance for CD163^+^. When both receptors were analyzed together, CD163^+^ CD169^−^ frequency was higher in PPM than PAM, whereas CD163^+^CD169^+^ cells were more prevalent in PAMs than in PPMs (Figure 1B). No significant differences were observed between the populations for CD163^−^CD169^+^ or CD163^−^CD169^−^ cells.

In terms of receptor expression levels, measured by mean fluorescence intensity (MFI), PAMs showed higher MFI for both CD163 and CD169, with the most pronounced difference observed in CD169 levels (Figure 1C). In contrast, PPMs exhibited higher MFI for CD14 than PAMs.

In conclusion, while PPMs express both CD163 and CD169, key receptors required for PRRSV infection, their frequency and intensity of expression were lower than in PAMs.

### 3.2 PPMs are highly susceptible to PRRSV infection

**Figure 2:**
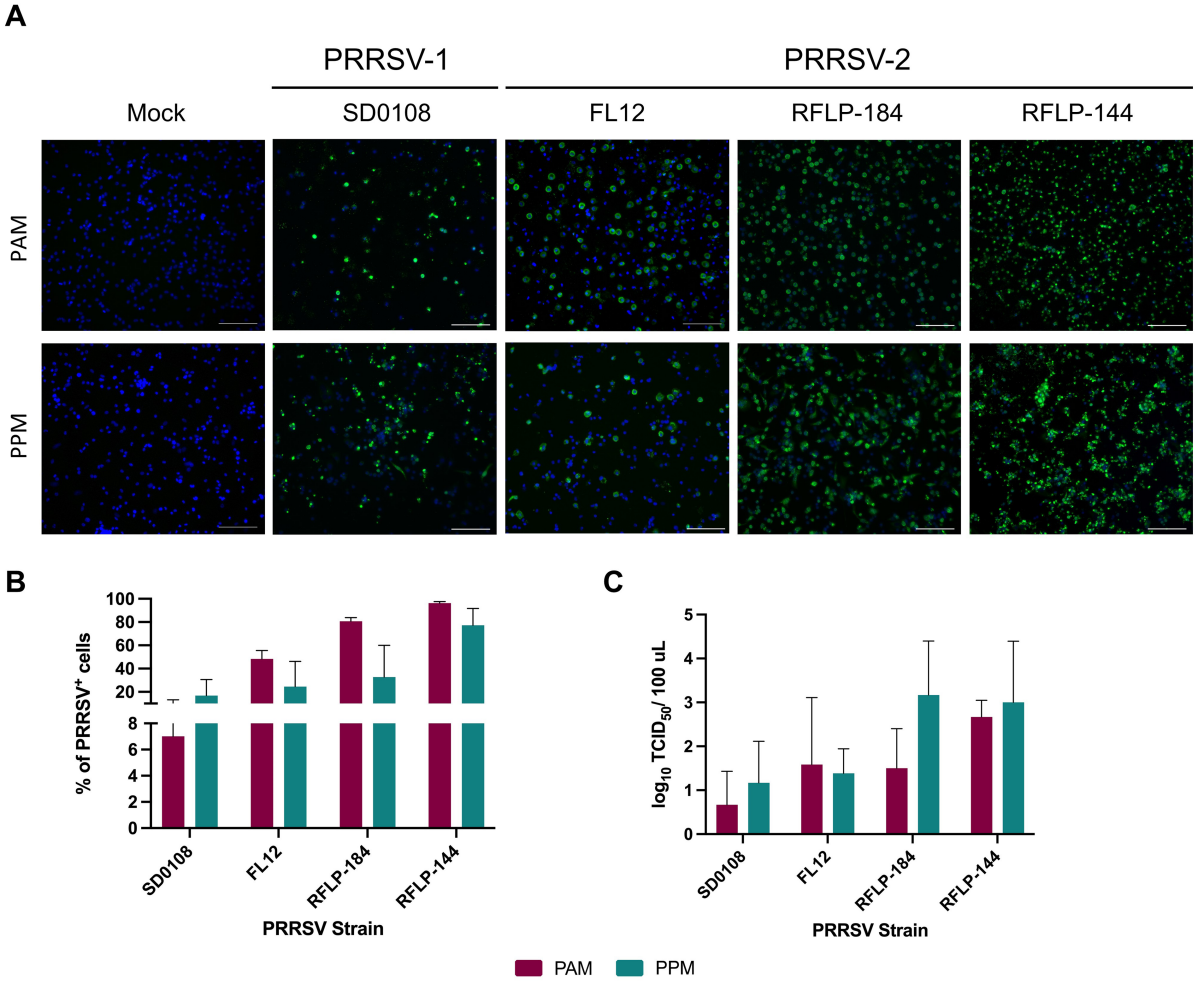
PPMs are susceptible to PRRSV infection. PAMs and PPMs were cultured *ex vivo* for 24 h. The cells were then infected with four different PRRSV strains at an MOI of 2. **(A)** At 24 hpi, cells were fixed and stained with the anti-N antibody to detect viral infected cells (shown in green). Cell nuclei were counterstained with DAPI (blue). Scale bar = 100 μm. **(B)** At 24 hpi, the frequencies of cells infected with PRRSV were analyzed by flow cytometry. Data are expressed as mean and standard deviation the percentage of PRRSV^+^ cells. **(C)** At 24 hpi, culture supernatants were collected, and infectious virus titers were measured in PAMs. Viral yield was determined by subtracting the virus titer at time 0 from the titer at 24 h. The experiments were conducted using cells collected from three different pigs.

To assess their susceptibility to PRRSV infection, PPMs and PAMs were cultured *ex vivo* for 24 h and then inoculated with a PRRSV isolate at an MOI of 2. At 24 h post-infection (hpi), virus-infected cells were detected using an IFA. A significant number of PRRSV^+^ cells were observed in PPM cultures infected with four different PRRSV isolates, indicating that these cells are susceptible to PRRSV infection (Figure 2A).

Next, we quantified the frequency of PRRSV^+^ cells using flow cytometry. The infection rates in PPM cultures varied depending on the virus isolate tested. Notably, PPM cultures inoculated with the two field isolates, RFLP-184 and RFLP-144, showed higher frequencies of PRRSV^+^ cells than those inoculated with SD0108 and FL12. Interestingly, no significant differences were observed in the frequency of PRRSV^+^ cells between PPMs and PAMs (Figure 2B).

To determine whether PPM cultures produced infectious viruses, supernatants were collected from infected cells at 24 hpi and titrated in PAMs to evaluate virus yield. For all four PRRSV isolates tested, PPM cells produced viral titers comparable to those observed in PAMs (Figure 2C). These findings collectively demonstrate that PPMs are susceptible to PRRSV infection.

### 3.3 Expression of CD163 and CD169 in PRRSV-infected cells

**Figure 3:**
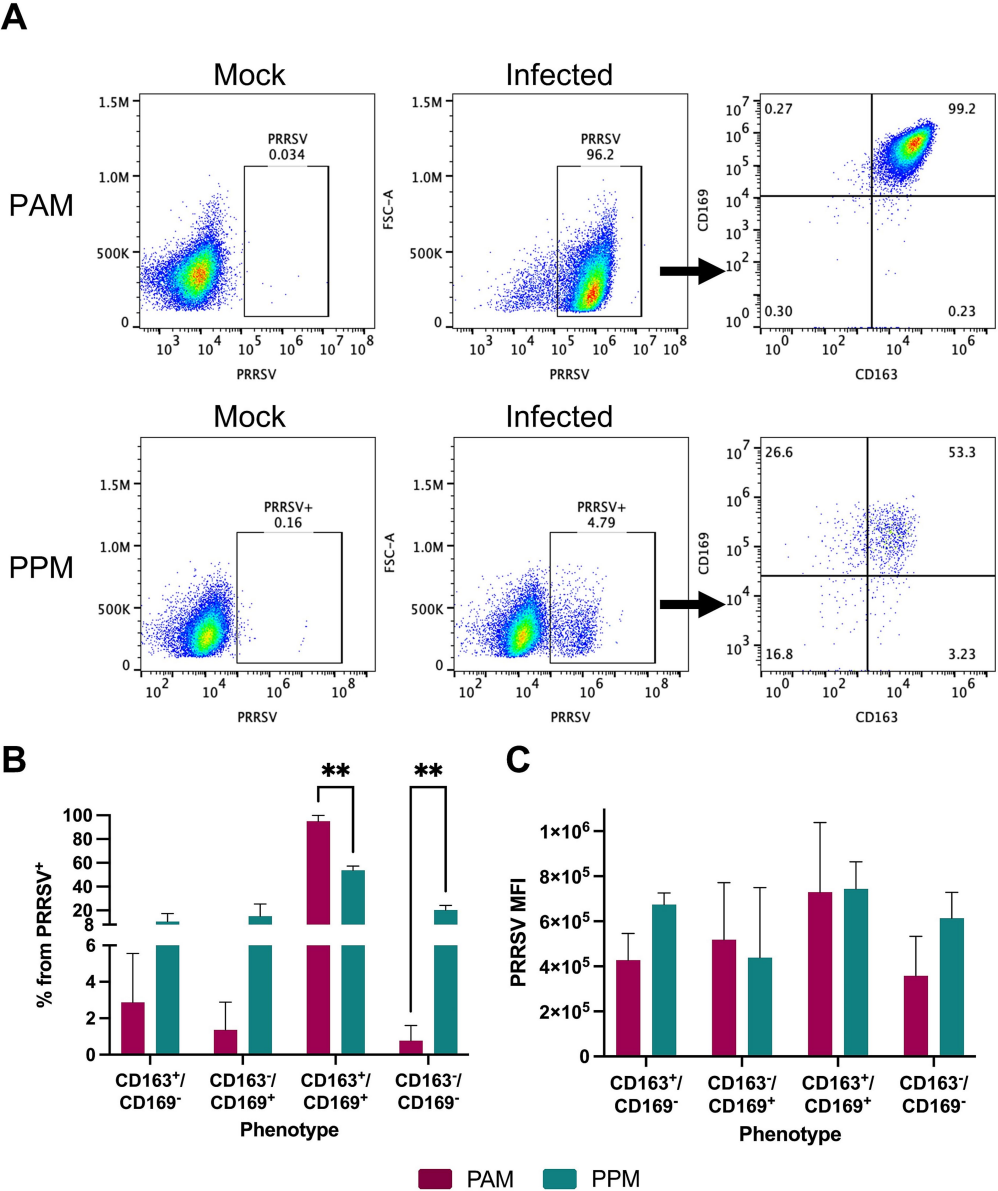
Expression of CD163 and CD169 in PRRSV-infected cells. Freshly isolated cells were infected with the PRRSV isolate RFLP-144 at an MOI of 2. At 24 hpi, cells were stained with antibodies against CD163, CD169, and PRRSV-N protein and analyzed by flow cytometry. Cells were first gated for PRRSV^+^ populations and subsequently analyzed for CD163 and CD169 expression within the PRRSV^+^ population. **(A)** Representative gating strategy. **(B)** Percentage of different cell populations within the PRRSV^+^ cells. **(C)** MFI of PRRSV-N protein expression in different cell populations. Experiments were performed using cells from three different pigs. ** *p* ≤ 0.01.

Previous studies have reported that PRRSV infection in PAMs leads to the downregulation of CD163 (Wahyuningtyas et al., 2021). To investigate whether this phenomenon also occurs in PPMs infected with PRRSV, we assessed the expression of CD163 and CD169 within the PRRSV^+^ population at 24 h post-infection (Figure 3A).

In both PAMs and PPMs, the majority of PRRSV^+^ cells continued to express both CD163 and CD169. However, in PPMs, a significant percentage of PRRSV^+^ cells was negative for both CD163 and CD169, and this population was larger compared to that observed in PAMs (Figure 3B). We found no significant differences in the MFI of PRRSV-N protein among PAM and PPM cell populations (Figure 3C). This suggests that the expression of CD163 and CD169 on the cell surface does not correlate with the intensity of PRRSV replication.

### 3.4 *Ex vivo* cultured PPM cells are more susceptible to PRRSV infection than freshly isolated cells

The susceptibility of macrophages to PRRSV infection is influenced by their state of differentiation and activation. Notably, PAMs cultured *ex vivo* for 24 h are more susceptible to PRRSV compared to freshly isolated PAMs (Duan et al., 1997b). In our previous experiment (Figure 2), we assessed the susceptibility of PPM after 24 h of *ex vivo* culture. To further explore the impact of cultivation on PPMs susceptibility, we compared freshly isolated PPMs with those cultured for 1 day.

**Figure 4:**
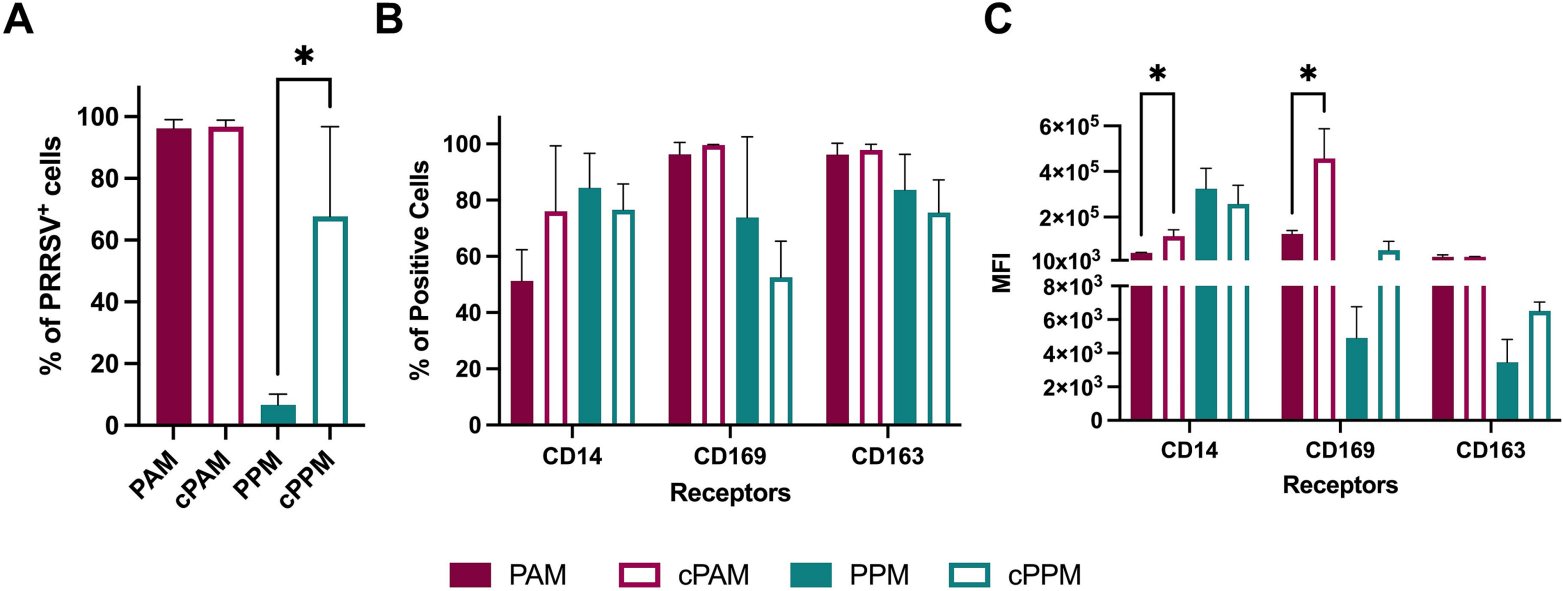
Cultured PPMs are more susceptible to PRRSV. Freshly isolated or 24-h cultured PPMs and PAMs were inoculated with the PRRSV isolate RFLP-144 at an MOI of 2. At 24 hpi, cells were analyzed for the expression of viral N protein and the cellular markers CD14, CD163, and CD169 by flow cytometry. **(A)** Frequency of PRRSV-infected cells. **(B)** Frequency of cells expressing the indicated cellular markers. **(C)** MFI of the indicated markers. The experiments were conducted using cells from three different pigs. PAM/PPM, Freshly isolated cells; cPAM/cPPM, Cells cultured for 24 h before infection. *p ≤ 0.05.

Freshly isolated PPMs were susceptible to PRRSV infection; however, the percentage of PRRSV^+^ cells in freshly isolated PPMs (6.7%) was significantly lower than in PPMs cultured for 24 h (67%) (Figure 4A). We did not observe such a difference in PAMs, as about 95% of the cells were PRRSV-positive under both conditions.

To determine whether the increased susceptibility in PPMs after 1 day of culture was associated with changes in macrophage phenotype, we assessed and compared the expression of CD14, CD163, and CD169 between freshly isolated and cultured cells. There were no significant differences in the percentage of cells expressing these markers in either PPMs or PAMs (Figure 4B). In PAMs, the MFI of CD14 and CD169 increased after 24 h of culture, while the MFI of CD163 remained unchanged. In PPMs, the MFI of CD163 and CD169 also increased after 24 h of culture but this increase was not statistically significant. Thus, the elevated susceptibility of PPMs to PRRSV infection after 24 h of *ex vivo* culture did not correlate with the changes in CD163 or CD169 expression.

### 3.5 Infection of PPMs is dependent of CD163

**Figure 5:**
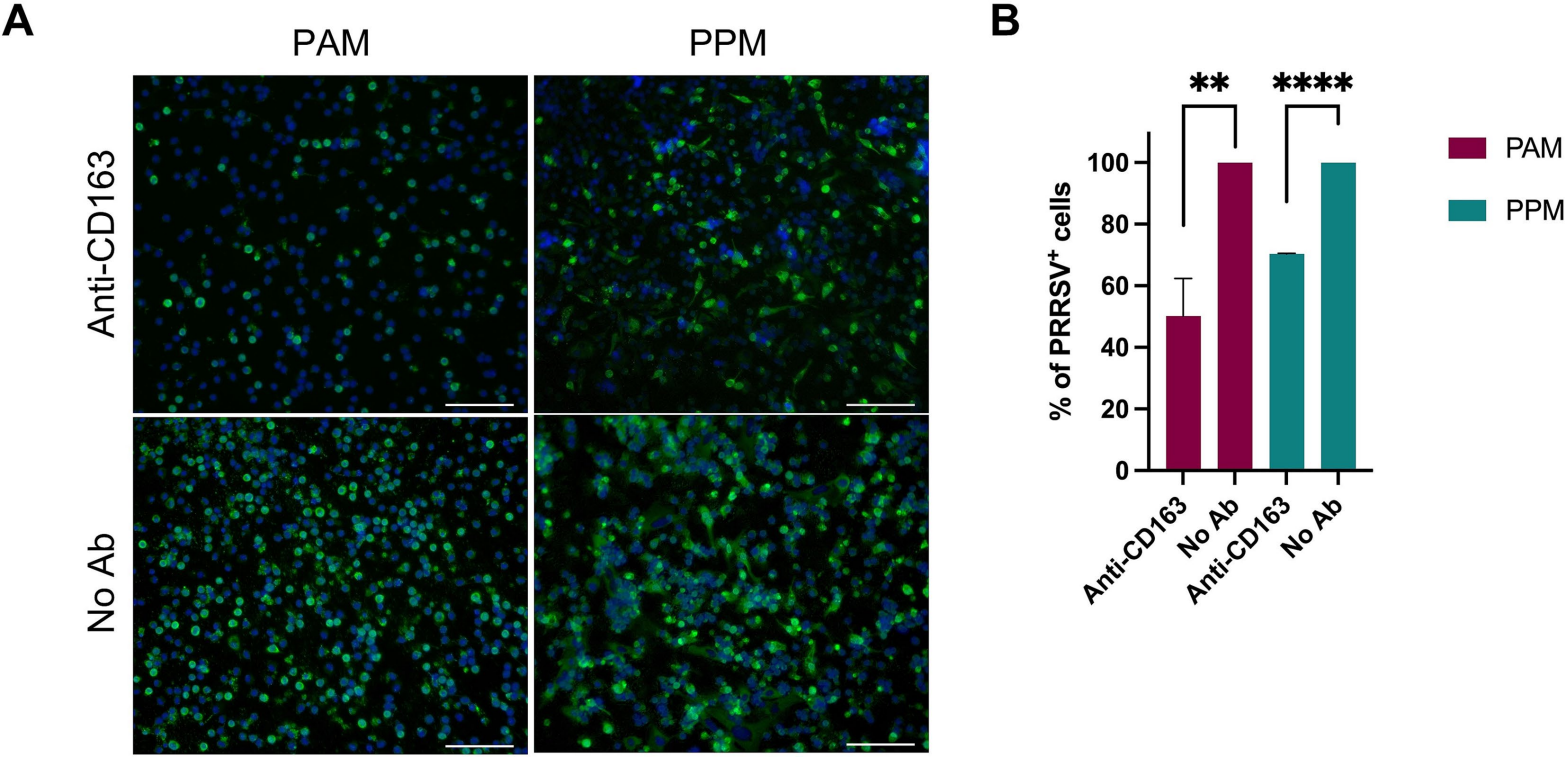
Infection of PPMs is dependent on CD163. PAMs and PPMs were cultured for 24 h and incubated with anti-human CD163 polyclonal antibody for 1 h prior to infection with PRRSV FL12 at an MOI of 2. Cells without antibody treatment (No Ab) were used as controls. At 24 hpi, cells were fixed and stained with an antibody specific to the viral N protein to detect infected cells. **(A)** Representative images showing PRRSV-infected cells (green). Cell nuclei were counterstained with DAPI (blue). Scale bar = 100 μm. **(B)** Percentage of PRRSV-positive cells determined by flow cytometry. The experiments were conducted using cells from three different pigs. **p ≤ 0.01, ***p ≤ 0.001.

CD163 is a key receptor for PRRSV infection (Calvert et al., 2007). To assess its role in PPMs’ susceptibility to PRRSV, we performed a receptor-blocking assay by pre-incubating the cells with a polyclonal antibody specific to CD163 before infection. We observed a significant reduction in the number of PRRSV-positive cells in both PAMs and PPMs treated with the anti-CD163 antibody compared to the control group (Figure 5A). However, the reduction was less pronounced in PPMs than in PAMs. Specifically, in PAMs, treatment with the anti-CD163 antibody led to a 50% reduction in PRRSV-positive cells, whereas in PPMs, the reduction was only 30% (Figure 5B).

Although the impact of CD163 blocking on PRRSV infection was smaller in PPMs, these results demonstrate that CD163 still plays a role in PRRSV susceptibility in PPMs, even if the dependence on CD163 is less compared to PAMs.

## 4 Discussion

In this study, we assessed the expression of CD163 and CD169 in PPMs and found that these receptors are expressed by the majority of cells, either individually (CD163^+^CD169^−^ or CD163^−^CD169^+^) or simultaneously (CD163^+^CD169^+^). The key difference between PAMs and PPMs is that PAMs exhibit a higher frequency of cells expressing CD169 compared to PPMs. Additionally, the intensity of CD163 and CD169 expression is higher in PAMs than in PPMs. These differences may explain why PAMs are the preferred target of PRRSV (Duan et al., 1997a). Notably, we detected CD169 expression in PPM cells, which contradicts previous findings (Duan et al., 1997b; Duan et al., 1998b). This discrepancy may be due to the difference in the mAb clones used for detecting CD169. Another major difference between PPMs and PAMs observed in this study is that PPMs expressed higher levels of CD14 and lower levels of CD163 and CD169 compared to PAMs. High expression of CD14 and low expression of CD163 and CD169 are associated with the immature phenotype of macrophages (Law et al., 1993; Mccullough et al., 1999; Sanchez et al., 1999; Hartnell et al., 2001; Chamorro et al., 2005). These results suggest that PPMs represent a more immature macrophage population compared to PAMs.

Previous studies have indicated that PRRSV preferentially infects differentiated, mature macrophages (Duan et al., 1997b). Particularly, the susceptibility of PAMs to PRRSV significantly increases after 1 day of *ex vivo* culture, whereas PPMs remain resistant to PRRSV infection even after culture (Duan et al., 1997b). We did not observe any significant difference in susceptibility to PRRSV between freshly isolated PAMs and those cultured for 1 day. This may be due to our use of a high multiplicity of infection, which resulted in 95% of the cells being infected and thus saturated the infection capacity. Different from the previous study, we found that PPMs are susceptible to infection by four PRRSV isolates, including one PRRSV-1 and three PRRSV-2 isolates, and that overnight culture of PPMs significantly enhanced the susceptibility of the cells to PRRSV infection. In this study, we inoculated PPMs with an MOI of 2, whereas the previous study used an MOI of 0.2. The differences in viral strain and MOI between the two studies may explain the contrasting results. The origin of the myeloid cells can also impact their permissiveness to PRRSV. For instance, plasmacytoid dendritic cells (pDC) do not support the complete replication cycle of PRRSV (Calzada-Nova et al., 2011; Bordet et al., 2018). Nonetheless, PPMs infected with all four tested PRRSV isolates produced infectious viruses, ruling out the possibility of incomplete infection. It has been observed that macrophages can sustain changes to adapt to culture conditions (Subramanian et al., 2022). Perhaps culturing PPMs triggered changes that affected their maturation or activation stage, leading to increased susceptibility. Interestingly, there were no significant differences in the expression levels of the markers CD14, CD163, and CD169 between freshly isolated PPMs and those cultured for 1 day (Figure 4C). Therefore, the increased susceptibility of cultured PPM to PRRSV infection cannot be attributed to changes in the expression of CD163 or CD169. The underlying reasons for the higher infection rate in PPMs cultured for 1 day before infection remain unknown at this time. Perhaps, comparing the gene-expression profiles between freshly isolated PPMs and cultured PPMs could help to identify changes in host factors that contribute to the enhanced susceptibility.

We observed a substantial number of PRRSV^+^ cells in both PPMs and PAMs that did not express CD163, CD169, or both markers. This finding is consistent with previous studies in PAMs or in blood monocytes treated with dexamethasone, where some PRRSV^+^ cells lacked CD163 expression (Sang et al., 2014; Singleton et al., 2018; Wahyuningtyas et al., 2021). Since we analyzed CD163 and CD169 expression after PRRSV infection, the presence of PRRSV^+^ CD163^−^ cells should not be interpreted as the virus infecting cells without CD163. Instead, it is likely that viral infection leads to the downregulation of CD163 expression.

Pre-treatment of PAMs and MARC-145 cells with anti-CD163 antibodies decreases their susceptibility to PRRSV infection in a dose-dependent manner. Additionally, cells with higher levels of CD163 require larger quantities of anti-CD163 antibodies to achieve complete infection blockade (Calvert et al., 2007). In the present study, we found that treating PAMs and PPMs prior to PRRSV inoculation significantly reduced the frequency of PRRSV^+^ cells, clearly indicating that their susceptibility to infection depends on CD163 expression. However, the reduction in infection was more pronounced in PAMs than in PPMs although PAMs and PPMs were treated with the same amount of anti-CD163 antibodies. This is interesting since PPM cells had significantly lower expression of CD163 (Figure 1C), thus, the expectation was that the reduction should be more profound in PPMs than in PAMs. Perhaps other unknown factors may contribute to the susceptibility of PPMs.

In summary, PPMs express both CD163 and CD169, the key cellular receptors for PRRSV infection, although the frequency of cells expressing these receptors, and the intensity of their expression are lower compared to PAMs. Notably, PPMs are susceptible to PRRSV infection, with susceptibility being dependent on CD163. The susceptibility of PPMs significantly increases after overnight culture, and this increase appears to be independent of CD163, CD169, or CD14 expression. This observation presents an opportunity to investigate other factors that may influence PRRSV infection.

## Data Availability

The original contributions presented in the study are included in the article/supplementary material, further inquiries can be directed to the corresponding author.
